# Erratum for “Proteinuria in dogs with gallbladder mucocele formation: A retrospective case control study”

**DOI:** 10.1111/jvim.16513

**Published:** 2022-08-04

**First published: 06 February 2021** https://onlinelibrary.wiley.com/doi/10.1111/jvim.16051

**Volume 35, Issue 2**

Abstract Methods section, first sentence, units are corrected as follows.

Retrospective case control study. Proteinuria defined by calculated urine dipstick protein concentration (**mg/dl**) to urine specific gravity (USG) ratio.

Section 2.3 Assessment of proteinuria, last paragraph, last sentence, units are corrected as follows and calculation of urine protein/urine specific gravity ratio of 1.5 was obtained by dividing the dipstick protein concentration (mg/dl) by the last 2 digits of the USG (30/20 = 1.5).

A cutoff for assuming abnormal protein concentrations in urine was defined as a dipstick protein‐to‐USG ratio of ≥1.5, which represents 1+ proteinuria (**30 mg/dl**) in urine with a USG of 1.020 as the upper limit of normal.^27^

